# Weighting Primary Care Patient Panel Size: A Novel Electronic Health Record-Derived Measure Using Machine Learning

**DOI:** 10.2196/medinform.6530

**Published:** 2016-10-14

**Authors:** Alvin Rajkomar, Joanne Wing Lan Yim, Kevin Grumbach, Ami Parekh

**Affiliations:** ^1^Department of MedicineUniversity of California, San FranciscoSan Francisco, CAUnited States; ^2^Clinical SystemsUniversity of California, San FranciscoSan Francisco, CAUnited States; ^3^Office of Population Health and Accountable Care, UCSF HealthUniversity of California, San FranciscoSan Francisco, CAUnited States; ^4^Department of Family and Community MedicineUniversity of California, San FranciscoSan Francisco, CAUnited States

**Keywords:** primary health care, risk adjustment, patient acceptance of health care, ambulatory care, health care economics and organizations, medical informatics, machine learning

## Abstract

**Background:**

Characterizing patient complexity using granular electronic health record (EHR) data regularly available to health systems is necessary to optimize primary care processes at scale.

**Objective:**

To characterize the utilization patterns of primary care patients and create weighted panel sizes for providers based on work required to care for patients with different patterns.

**Methods:**

We used EHR data over a 2-year period from patients empaneled to primary care clinicians in a single academic health system, including their in-person encounter history and virtual encounters such as telephonic visits, electronic messaging, and care coordination with specialists. Using a combination of decision rules and k-means clustering, we identified clusters of patients with similar health care system activity. Phenotypes with basic demographic information were used to predict future health care utilization using log-linear models. Phenotypes were also used to calculate weighted panel sizes.

**Results:**

We identified 7 primary care utilization phenotypes, which were characterized by various combinations of primary care and specialty usage and were deemed clinically distinct by primary care physicians. These phenotypes, combined with age-sex and primary payer variables, predicted future primary care utilization with *R*^2^ of .394 and were used to create weighted panel sizes.

**Conclusions:**

Individual patients’ health care utilization may be useful for classifying patients by primary care work effort and for predicting future primary care usage.

## Introduction

In the face of increasing demand for primary care services [1] and concerns of a primary care physician (PCP) shortage [2], health systems need methods to effectively match primary care workload and capacity [3]. Empanelment, assigning each patient to a primary care physician (PCP) or team, is an essential building block for high-performing primary care [4,5].

Health systems moving toward empaneled models of care must account for the truism that no two patients are the same; different patients require substantially different amounts of primary care work effort to address their health care needs [3]. Methods are needed to acknowledge and predict how much primary care work effort a patient needs in order to adjust panel sizes to account for differences in patient mix across individual PCPs and practices to better match capacity with demand. The methods could also be used to adjust panel-based payment to pay a higher capitated rate for patients requiring more primary care work effort.

Traditional methods to adjust panel size using basic patient demographic data such as age and sex have limited predictive power [6]. These approaches have been augmented by other approaches that are limited by requiring multiple data sources (eg, pharmacy data and insurance claims), poor utility in predicting primary care work effort, their proprietary natures, and lack of validation in the literature [7-11].

The lack of a validated predictive model and the desire of our academic health system to use case-mix–adjusted primary care physician (PCP) panel sizes in our own operations motivated us to use machine learning methodologies on regularly collected electronic health record (EHR) data to create a novel method to adjust panel sizes. Given the variety of diagnoses possible in a population and the spectrum of care complexity for different patients with the same diagnoses, the phenotypes in our model are based on objectively measured interactions with the health system rather than on disease-based codes entered by clinicians in the EHR. In this paper, we describe our method of using patient utilization phenotypes to better characterize primary care work effort to develop a novel methodology for weighting primary care panel size.

## Methods

### Overview of Study Design

Our overall study design consisted of 3 major steps.

Define utilization phenotype clusters: We used a training set sample of patients with year 1 EHR data on health system encounters to cluster patients into distinct utilization phenotypes, using k-means clustering methods.

Validate utilization phenotype clusters: We determined among patients in a separate test set if models using utilization phenotype clusters were better at predicting year 2 primary care visits than models using simpler, raw counts of year 1 encounters, using log-linear regression models.

Determine weights for each phenotype cluster for computing weighted panel sizes: We consolidated utilization phenotype clusters into a smaller number of final primary care work clusters, weighted each final cluster based on median number of concurrent year primary care visits among patients in each cluster, and applied these weights to the entire sample of empaneled patients.

### Sample and Data Sources

We used the EHR system (Epic, Madison, WI, USA) to collect data on all patients older than 18 years empaneled as of January 31, 2015, to a primary care clinician in practices operated by the University of California, San Francisco health system (UCSF Health). Empanelment at UCSF Health is defined as having an identified UCSF Health primary care clinician listed in the EHR primary care provider field and at least 1 visit in the prior 3 years to any clinician at the primary care practice; 52,368 adult patients were empaneled at primary care practices in January 2015.

**Figure 1:**
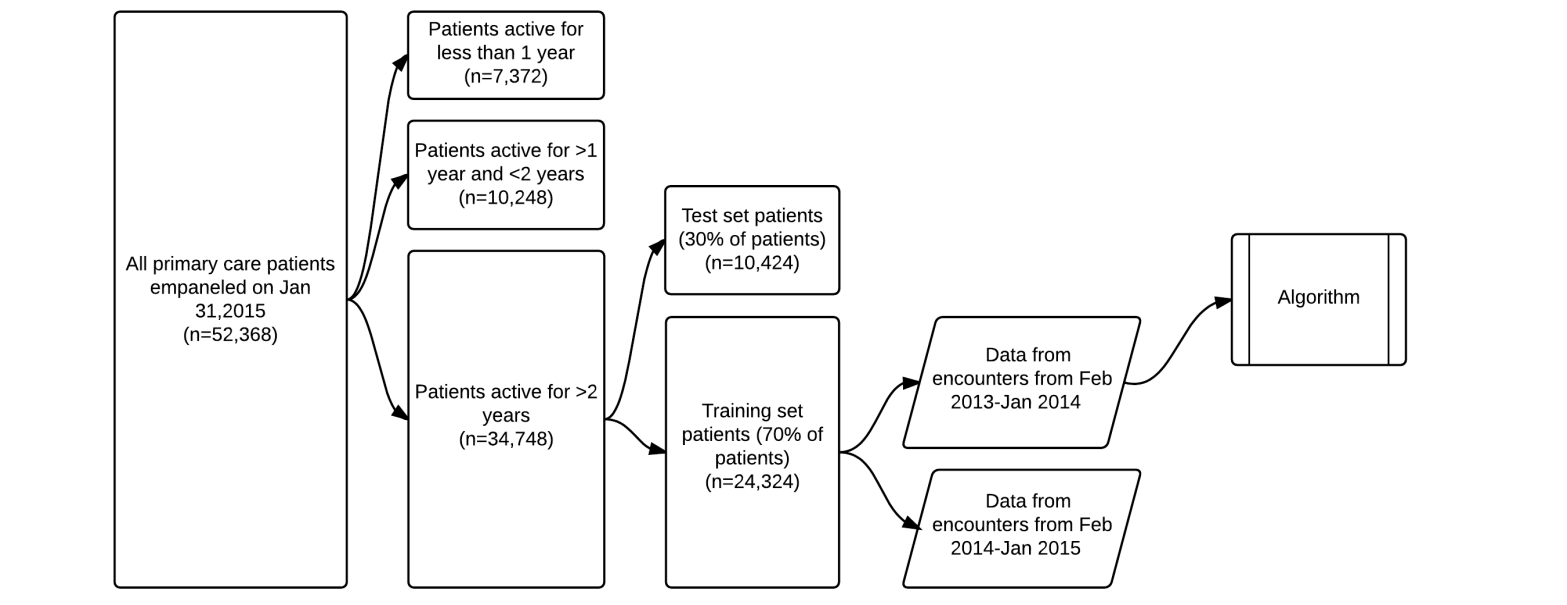
Flowchart of data from the electronic health record to the algorithm. PCWC: Primary care work cluster.

**Figure 2:**
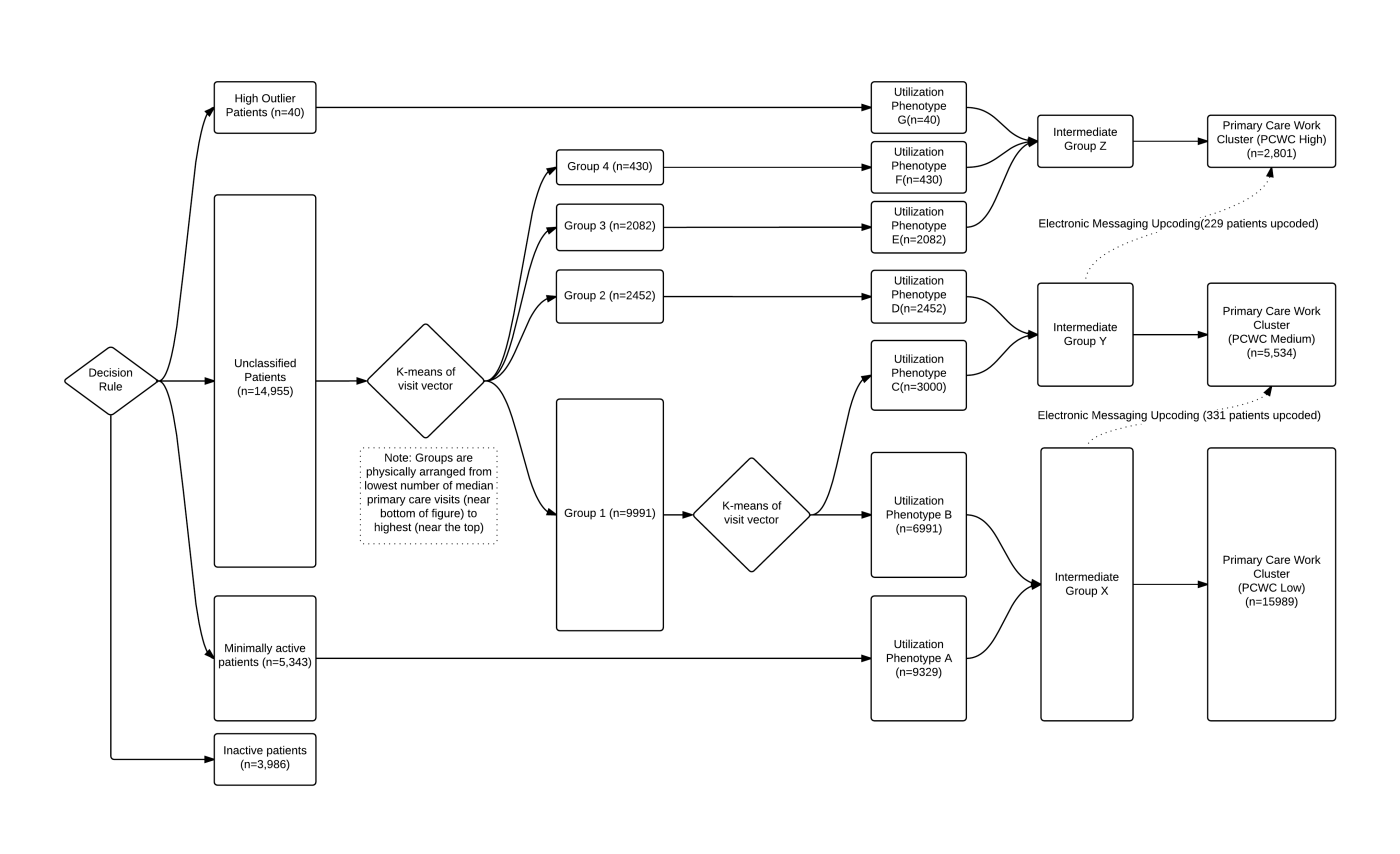
Flowchart of decision rules and clustering algorithms that demonstrate how patients were classified into different utilization phenotypes and primary work group clusters.

For model development, we included only the subset of 34,748 patients who had at least 1 encounter (including office visit, telephone, electronic messaging, or medication refill) occurring on or before February 1, 2013, to ensure that patients in the study would have at least 12 months of eligibility for data analysis for deriving the predictive model (February 1, 2013 to January 31, 2014) and then a subsequent 12 months of data for using the model to predict utilization (February 1, 2014 to January 31, 2015). The model was developed on a training set of a random sample of 70.00% (24,324/34,748) of these patients, and the remaining 30.00% (10,424/34,748) were left as a test set (Figures 1 and 2).

### Variables Used

#### Variables Included in Weighting Algorithm

For each patient, we retrospectively collected the data for the following types of encounters at our health system between July 1, 2012 and January 31, 2015: primary care office visits billed for more than 5 minutes, missed appointments, emergency department visits, emergent hospitalizations, elective hospitalizations, infusion and transfusion center visits, medical and surgical subspecialty visits, diagnostic and interventional radiology visits, telephone encounters with any member of their assigned primary care team, urgent care visits, and electronic messages with their primary care team through the EHR secure messaging system. In addition, we collected demographic data including age, sex, race-ethnicity, primary payer, primary care clinic location, and primary care clinician. We also included every medication documented in the EHR medication list, including start and stop dates.

For each patient in the training set, we created a visit vector that represented the various encounters across the health system. Each component of the vector was created by summing the total number of visits within a respective encounter type that occurred from February 1, 2013 to January 31, 2014. The encounter types included “effective number” of primary care visits (an adjusted visit count incorporating medication counts, as defined below), telephone encounters with the primary care office, missed appointments to the primary care office, urgent care visits, emergency room visits, emergent hospitalizations, routine hospitalizations, medical and surgical specialty visits, infusion center visits, transfusion center visits, diagnostic and interventional radiology visits, and electronic messaging. Other than primary care and specialty visits, each encounter was an equal contributor to its respective category.

We created an effective number of primary care visits to account for additional time required for medication reconciliation and complexity of PCP visits for patients with multiple medications. For each visit, we calculated the number of active medications. If there were 5 or fewer active medications at that particular primary care visit, then that visit was assigned a weight of 1. If there were 6 to 10 medications, then the visit was assigned a weight of 1.5. If the medication count was greater than 10, then the visit was assigned a weight of 1.75. The “effective” primary care office visit count was the sum of these weighted visits.

Some specialists care for diseases that require frequent visits, such as weekly dermatologic treatments, and other specialists may often monitor diseases that require only yearly follow-ups. Because of the high standard deviation of visit counts per year for different specialties, we capped the total number of visits counted for each specialty. The cap was set for each specialty separately at 2 standard deviations above the mean number of visits per year among all patients seen by that specialty. For example, if a patient had 20 dermatology visits and 2 cardiology visits, the total number of specialty visits we counted was 17.8 because the cap for dermatology visits was 15.8 and for cardiology visits 6.7.

#### Additional Variables Included in Algorithm Validation

For validation of the algorithm, we also included age, sex, and primary payer. Patients were split by age and sex into 12 categories, using the age groups 18-34, 35-49, 50-64, 65-69, 70-84, and 85-115 years. The 3 patients with missing sex were categorized as female in order to keep the patients in the analytic sample. The primary payers were characterized as commercial, Medicare, Medicaid, or other.

### Primary Care Focus Group for Expert Consensus

As we refined the algorithm, we asked a focus group of practicing PCPs to qualitatively evaluate whether the clusters our methodology identified aligned with their perception of the level of work needed for their patients. The group included 15 family physicians and general internists.

### Algorithm

**Figure 3:**
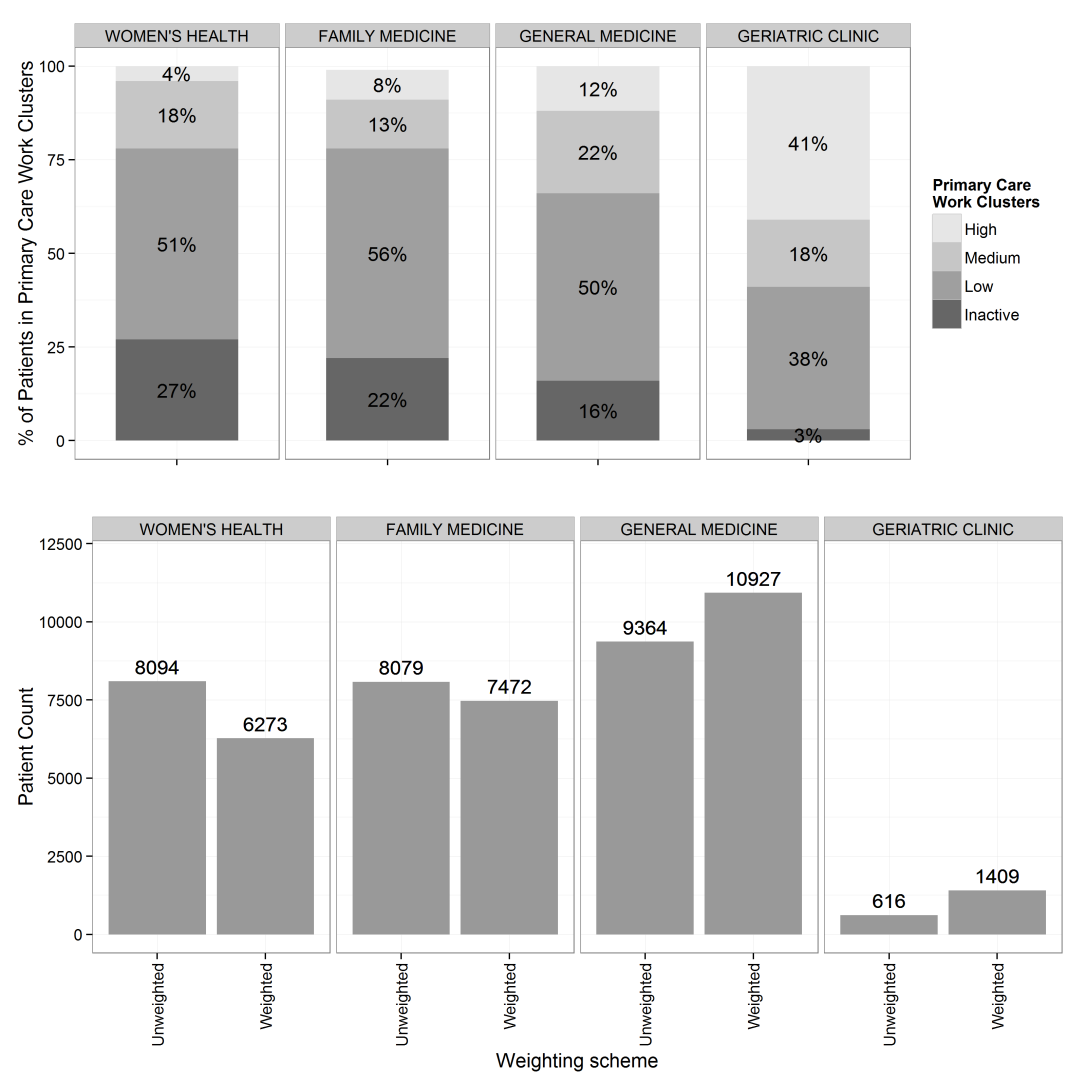
The fractions of all patients assigned to the primary care work clusters in 4 selected clinics and their unweighted and weighted panel sizes. The distribution of patients across clusters was unique to each clinic, and because each cluster is weighted differently, the difference between weighted and unweighted panel sizes differed for each clinic as well. The geriatric clinic, which has 41% of its population assigned to the high work cluster, had a weighted panel size that was more than twice the unweighted size.

The algorithm was developed using only the patients in the training set. We used a decision rule for initial classification of patients (Figure 3). Patients with greater than 6 standard deviations above the mean number of annual primary care visits were classified as “high outliers.” Patients who met all the following criteria were classified as “minimally active”: ≤1 primary care visit per year, 0 emergency department visits, 0 hospitalizations, ≤4 specialty visits per year, ≤2 telephone encounters per year, and ≤6 electronic messages to the patient per year. However, if patients had zero visits across all these categories (excluding missed appointments), then they were classified as “inactive patients.” Only patients not meeting the criteria for “high outliers,” “minimally active,” and “inactive patients” entered the next stage of the algorithm. In the algorithm, these patients were divided into 4 groups by k-means clustering on the encounter vectors. At this point, all variables were of the same unit of analysis (eg, number of visits per year), which made the clusters easier to interpret. The selective truncation of some of the visit types as described in the Variables Included in Weighting Algorithms section was utilized in place of blindly normalizing by mean and standard deviation. All encounter categories except for electronic messaging were used in this step. The k-means clustering was performed using the Hartigan-Wong algorithm. We used 4 centers with 5 random initiations and up to a maximum of 10 iterations to find stable cluster definitions. We chose to use 4 clusters by examining the change in reduction of the within-group sum of squares (Multimedia Appendix 1) and by verifying with clinicians that their own patients assigned to the clusters were meaningfully distributed (see below).

The clusters were then ranked by the median annual number of raw PCP visits (ie, visit counts that were not weighted for number of medications). Our primary care physician (PCP) focus group decided that the cluster with the fewest visits contained 2 heterogeneous groups after examining the assignments of their own patients. Therefore, that cluster was further divided in 2 by k-means clustering, which aside from the number of clusters used the same algorithm and settings as the previous clustering (Figure 3).

Excluding the inactive patients, there were 7 resulting groups: 2 from the initial decision rules, 3 from the initial cluster assignment, and 2 from the second round. These 7 cluster groups represented different patterns of health care utilization across the health system—health care utilization phenotypes—which we labeled A through G (Figure 3).

The focus group of PCPs agreed that the groupings represented distinct primary care phenotypes but believed that some of the phenotypes required a similar amount of primary care work effort. Therefore, we collapsed the 7 phenotypes into 3 categories—intermediate groups X, Y, and Z (Figure 3), ranked by the median number of primary care visits per year among patients in the group.

A final decision rule was applied to account for patients’ use of secure electronic messaging with their providers. Patients who sent more than 1.5 standard deviations of electronic messages relative to the mean of all patients in the originally assigned category or who were sent more than 24 messages by their primary care clinician were moved to the next higher cluster. The final clusters were labeled high, medium, and low to represent the relative amount of primary work effort for patients in that cluster, with a fourth cluster being the inactive patients. Patients initially classified as “minimally active” were added to the “low” group. We refer to these as primary care work clusters.

### Validation

The utilization phenotypes were designed to cluster patients based on utilization patterns in a nonhypothesis-driven way. To demonstrate that the clusters had predictive power, we sought to validate them as part of a risk adjustment model predicting subsequent primary care service utilization. We created a series of generalized linear models to predict the total number of primary care encounters (PCP visits and telephone encounters) for each patient in the second year (February 1, 2014 to January 31, 2015). The models were developed using the same patients in the training set sample. As predictors, we used age-sex categories, payer type, and one of two variables measuring utilization patterns during the first year (February 1, 2013 to January 31, 2014): the 7 primary care utilization phenotypes (which we have described above) or a simpler measure of the raw counts of all types of encounters (which we refer to as the “naïve phenotype”). We used the 7 utilization phenotypes rather than the 3 work clusters, which are derived from the phenotypes, because the phenotypes were felt to encode meaningful clinical distinctions by the primary care focus group. The naïve phenotype was created by summing the total number of all in-person encounters (primary care visits, emergency department visits, hospitalizations, infusion and transfusion visits, urgent care visits, specialty visits, and outpatient procedures for cardiology, radiology, pulmonology, and neurology). These sums were rank ordered and divided into 7 percentiles so as to have the same number of categories as the primary care work clusters.

We then applied the coefficients derived from the training set to predict the log number of primary care visits in the second year for the test set of the sample, which was not used to generate the model. We report the adjusted *R*-squared and the Akaike information criterion (AIC). The AIC is a goodness-of-fit value that balances model bias versus variability, ranges from 0 to infinity, and penalizes models with more variables.

We repeated the analysis with the outcome of the number of primary care visits only (not including telephone encounters). We also repeated the analysis modeling the raw rather than log number of visits per year with a Poisson and zero-inflated Poisson distribution with a canonical log link.

### Weighted Panel Size

The work clusters were used to calculate weighted panel sizes as of February 1, 2015, using all 52,368 adult patients empaneled in primary care. We assigned patients to 4 primary care work clusters using the algorithm defined by the training set, as described above, based on EHR data on activity at our health system between February 1, 2014 and January 31, 2015. For patients with less than 12 months of activity, we initially weighted the number of visits by the number of months the patient had an active status, but this gave patients with just a few visits with a short exposure time high counts in their visit vector (eg, 2 visits in 3 months would be calculated to an average of 8 visits per year). Instead, for those patients we assumed their visits were over 12 months.

Once patients were assigned to a primary care work cluster, we needed to assign weights to each of the 4 final clusters (high, medium, low, and inactive). In consultation with our focus group of clinicians, we decided to base the weights on the number of effective primary care visits among patients in each of the clusters between February 1, 2014 and January 31, 2015. The relative weights of the “medium” and “high” clusters were defined by dividing the median number of effective primary care visits among patients in each of these clusters by the median number of effective primary care visits in the “low” cluster. Because patients in the “inactive” cluster had no activity in the preceding 12 months but were still empaneled in primary care and might be expected to have some future activity, we assigned patients in the inactive cluster a weight of 0.05.

**Figure 4:**
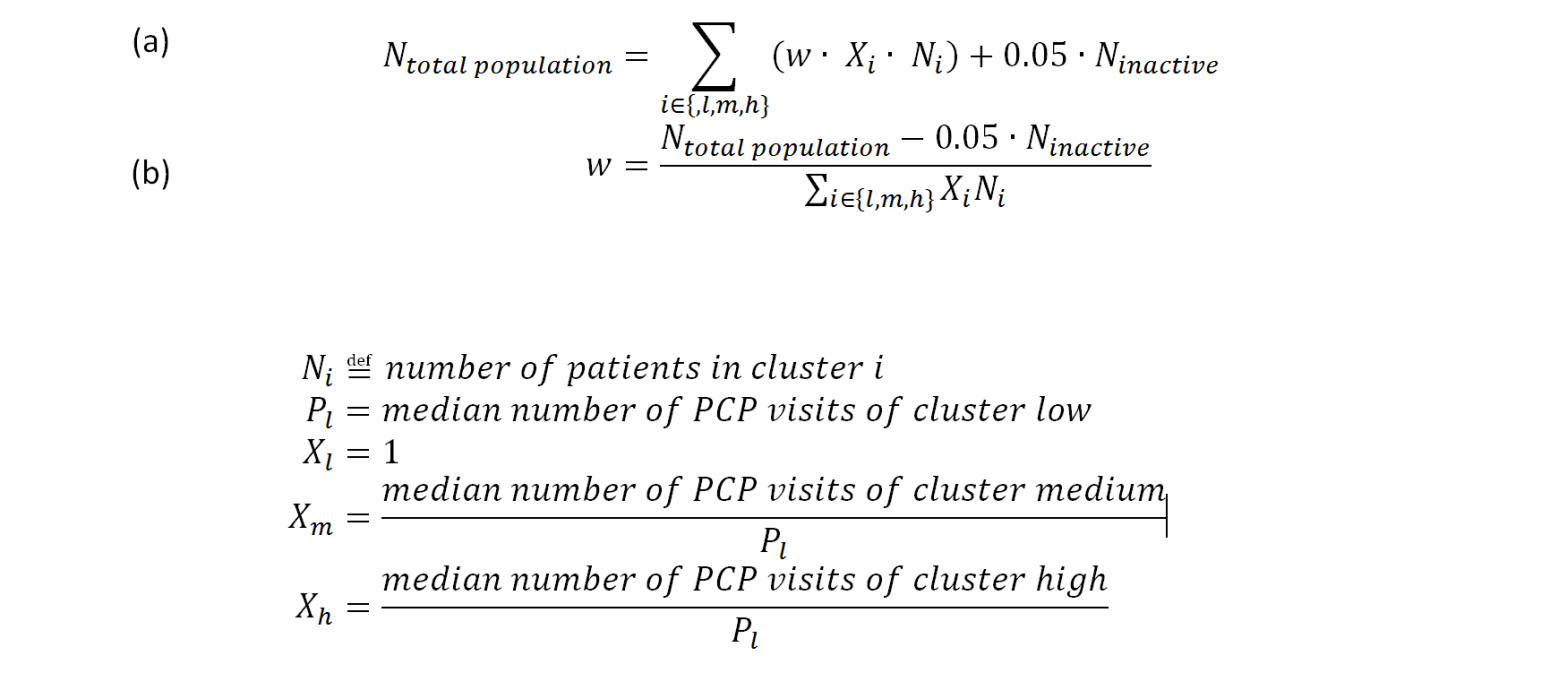
Equations that define how scaling factor *w* was defined. We constrain the total weighted population size (the right hand side) to be equal to the total unweighted population size in (a). We solve for *w* in (b) PCP: primary care physician.

Finally, to make the total number of weighted patients equal the total number of raw, unweighted patients empaneled in primary care (ie, 52,368), we used an additional scaling factor, *w*, to impose this restriction. (Figure 4)

The cluster weights for the low, medium, and high clusters were then defined to be *w* multiplied by the median number of PCP visits of the respective cluster divided by the median number of patients in the low cluster (Figure 4). To calculate an effective panel size for a clinic or primary care provider, each patient in the panel was classified to a primary care work cluster. The number of patients in each cluster was multiplied by the respective weight, and the sum over all clusters defined the weighted panel size.

To demonstrate how panel sizes for PCPs changed from the raw panel size to the weighted panel size, we calculated the average change in panel size. In this analysis, we only included PCPs who had an unweighted panel size of greater than 150 active patients.

All analyses were performed using R version 3.1.2 (R Foundation for Statistical Computing). The k-means algorithm was from the standard “stats” package (version 3.2.1). The research was approved by the Institutional Review Board at UCSF.

## Results

### Description of the Utilization Phenotypes and Primary Care Work Clusters

Of the 52,368 adult patients empaneled on January 31, 2015, a total of 34,748 were active for more than 2 years. Those were further subdivided into training and test sets of 24,324 and 10,424 patients (Figures 1 and 2). Characteristics of the patients in the training set and their utilization are presented in Tables 1 and 2.

Of the patients in the training set, 3986 were determined to be inactive, 5343 minimally active, and 40 high-outlier patients. The remaining 14,955 patients were k-means clustered based on the visit vector into 5 utilization phenotypes. These phenotypes were combined with minimally active and high-outlier patients into 7 phenotypes, which were further merged into 3 primary care work clusters (Figure 3).

The characteristics of patients in each utilization phenotype are presented in Tables 1 and 2 (Full table is in Multimedia Appendix 2). None of the demographic variables demonstrated a monotonic increase or decrease across the phenotypes, although phenotypes E-G tended to represent older, female, patients with government health plans.

**Table 1:** Patient characteristics of each utilization phenotypes (inactive through group D) in the training set (N=24,324).

Characteristics	Utilization phenotype				
	Inactive	A	B	C	D
Size of group (n)	3986	5343	6991	3000	2452
Age, years, mean (SD)	41.9 (17.3)	47.7 (14.7)	53.7 (16.8)	56.6 (16.4)	59.9 (17.3)
Male, n (%)	1551 (38.9)	2057 (38.5)	2678 (38.3)	1083 (36.1)	922 (37.6)
White, n (%)	1814 (45.5)	2875 (53.8)	3293 (47.1)	1635 (54.5)	1324 (54)
Asian, n (%)	694 (17.4)	1095 (20.5)	1734 (24.8)	675 (22.5)	596 (24.3)
Black, n (%)	379 (9.5)	289 (5.4)	587 (8.4)	222 (7.4)	184 (7.5)
Commercial, n (%)	2738 (68.7)	4266 (79.9)	4348 (62.2)	1731 (57.7)	1113 (45.4)
Medicare or Medicaid, n (%)	1068 (26.8)	992 (18.6)	2545 (36.4)	1245 (41.5)	1324 (54.0)
Other payer, n (%)	180 (4.5)	85 (2)	98 (1)	24 (1)	15 (1)
Active medications at PCP^a^ visit, mean (SD)	0 (0)	2.3 (2.9)	5 (3.6)	5.5 (4.3)	8.1 (6)
Primary care visits, mean (SD)	0 (0)	0.7 (0.5)	2.6 (1.3)	2.1 (1.4)	2.9 (2.3)
Weighted primary care visits, mean (SD)	0 (0)	0.7 (0.6)	3.2 (1.8)	2.8 (1.9)	4.3 (3.7)
No-show visits, mean (SD)	0.1 (0.4)	0.2 (0.7)	0.5 (1)	0.6 (1.2)	1.4 (2.1)
Urgent care visits, mean (SD)	0 (0)	0.1 (0.5)	0.2 (0.5)	0.2 (0.6)	0.2 (0.6)
Telephone encounters, mean (SD)	0 (0)	0.4 (0.7)	1.7 (1.8)	1.4 (1.6)	2.3 (2.5)
Emergency department visits, mean (SD)	0 (0)	0 (0)	0.2 (0.5)	0.2 (0.5)	0.3 (0.7)
Emergent hospitalizations, mean (SD)	0 (0)	0 (0)	0 (0.2)	0 (0.3)	0.1 (0.5)
Elective hospitalizations, mean (SD)	0 (0)	0 (0)	0 (0)	0 (0.2)	0.1 (0.3)
Specialist visits (capped), mean (SD)	0 (0)	1 (1.2)	1 (1)	5.5 (1.4)	14 (5.3)
Infusion visits, mean (SD)	0 (0)	0 (0.5)	0 (0.4)	0.1 (1)	0.7 (4.1)
Transfusion visits, mean (SD)	0 (0)	0 (0.8)	0 (0.2)	0.1 (0.8)	0.4 (2.6)
Radiology or procedure visits, mean (SD)	0 (0)	0.4 (0.8)	0.6 (1)	1.2 (1.4)	2.2 (2.6)
Secure electronic messages to patient, mean (SD)	0 (0)	0.7 (1.4)	2.3 (4.3)	4 (6.4)	6.8 (11.1)
Secure electronic messages from patient, mean (SD)	0 (0)	0.9 (1.8)	2.8 (5.3)	5 (8.2)	8.9 (15)

^a^PCP: primary care physician.

**Table 2:** Patient characteristics of each utilization phenotype (group E to G) in the training set (N=24,324). The total column includes data from phenotypes in Table 1.

Characteristics	Utilization phenotype			
	E	F	G	Total sample
Size of group (n)	2082	430	40	24,324
Age, years, mean (SD)	65.1 (16.8)	67.4 (16.3)	60.5 (14.4)	52.7 (17.9)
Male, n (%)	716 (34.4)	158 (36.7)	8 (20)	9170 (37.7)
White, n (%)	799 (38.4)	191 (44.4)	14 (35)	11,943 (49.1)
Asian, n (%)	525 (25.2)	76 (18)	4 (10)	5400 (22.2)
Black, n (%)	385 (18.5)	102 (23.7)	17 (43)	2165 (8.9)
Commercial, n (%)	431 (20.7)	26 (6)	3 (8)	14,665 (60.3)
Medicare or Medicaid, n (%)	1628 (78.2)	402 (93.5)	37 (93)	9219 (38.0)
Other payer, n (%)	23 (1)	2 (1)	N/A^a^	440 (1.8)
Active medications at PCP^b^ visit, mean (SD)	11 (5)	15.7 (6.1)	16.2 (9.3)	4.7 (5.2)
Primary care visits, mean (SD)	7 (2.8)	11.5 (4.5)	33.2 (10)	2.3 (3)
Weighted primary care visits, mean (SD)	10.7 (4.4)	19.1 (7.8)	53.1 (15.6)	3.2 (4.7)
No-show visits, mean (SD)	1.8 (2.4)	4.3 (4.9)	6.2 (5.3)	0.7 (1.6)
Urgent care visits, mean (SD)	0.2 (0.7)	0.5 (1.2)	1.2 (2.4)	0.1 (0.5)
Telephone encounters, mean (SD)	5.9 (3.8)	19.4 (10.3)	18.5 (22.5)	1.9 (3.8)
Emergency department visits, mean (SD)	0.5 (1)	1.6 (2.9)	1.8 (2.1)	0.2 (0.7)
Emergent hospitalizations, mean (SD)	0.2 (0.5)	0.9 (1.7)	0.9 (1.5)	0.1 (0.4)
Elective hospitalizations, mean (SD)	0 (0.2)	0.1 (0.4)	0 (0.2)	0 (0.1)
Specialist visits (capped), mean (SD)	4.4 (3.3)	11.5 (8.2)	7.6 (9.2)	3.2 (4.9)
Infusion visits, mean (SD)	0.1 (2.2)	0.1 (1.1)	0 (0.2)	0.1 (1.5)
Transfusion visits, mean (SD)	0 (0.6)	0.5 (3.3)	0.2 (1.3)	0.1 (1.1)
Radiology or procedure visits, mean (SD)	1.5 (1.7)	2.7 (3)	2.5 (2.9)	0.8 (1.5)
Secure electronic messages to patient, mean (SD)	3.4 (7.8)	5.6 (14.6)	5 (14.5)	2.4 (6.1)
Secure electronic messages from patient, mean (SD)	4.4 (10.4)	8.1 (22.3)	8.6 (25.5)	3.1 (8.1)

^a^N/A: not applicable.

^b^PCP: primary care physician.

Patients with utilization phenotype A saw their primary care physician (PCP) less than once a year and tended not to have much health care exposure across the health system. Patients with phenotypes B, C, and D had a mean of 2 or more visits a year with their PCPs, although those with C had more than 5 times the average number of specialty visits compared with those with B, and D had 14 times more. Phenotypes E and F saw their PCPs on average more than 7 times a year, with phenotype F also having more than double the number of specialty visits compared with E. Phenotype G was predefined as the “high-outlier” group and saw their primary care doctor on average more than 30 times a year.

The characteristics of patients in each of the final 4 primary care work clusters, which were created by combining utilization phenotypes, are provided in Multimedia Appendix 3. The medium and high clusters tended to be older and female and to have Medicare or Medicaid. There was a monotonic increase of nearly every component of the encounter vector except for specialist visits and infusion center visits.

### Validation of the Prediction of Primary Care Office and Telephone Visits

The results of the linear models to predict log-transformed primary care office and telephone visits are presented in Table 3. A model with only age-sex and payer accounted for 20.9% of the variance of primary care office and telephone visits the next year. When we added the naïve phenotype, or groupings based on the raw number of total in-person health care encounters as described above, 34.4% of the variance was captured by the model. If the utilization phenotype was used instead of the naïve phenotype, 39.4% of the variance was modeled. This model had the lowest AIC, which indicates a better fit even accounting for additional variables in the model. The results were similar with generalized linear models of a zero-inflated Poisson regression predicting unlogged counts, which are not shown.

We also report the results of a similar model predicting just the office-based primary care visits (Multimedia Appendix 4) where the age-sex group, payer, and utilization phenotype demonstrated the best fit of the data with 34.4% of the variance of log of visit number captured.

### Weighted Panel Sizes

Using the entire sample, we calculated the weights for the different primary care work clusters using concurrent year primary care visits to determine weights, as described above. The inactive, low, medium, and high clusters had weights of 0.050, 0.659, 1.319, and 4.396, respectively.

Whereas the unweighted sizes of the inactive and low clusters were 11,830 and 26,091, the weighted sizes of these populations decreased to 591 and 17,205, respectively. Conversely, the weighted sizes of the medium and high clusters increased from 9404 to 12,402 and from 5043 to 22,169, respectively (Multimedia Appendix 5). By definition, the total unweighted population size was equal to the weighted population size.

Different clinics and PCPs had different proportions of patients in high, medium, and low clusters. Illustrative results for 4 primary care clinics caring for adults are displayed in Figure 3. Patients in the high and medium clusters combined constituted slightly more than 20% of adult patients at the Women’s Health Primary Care and Family Medicine Clinics, compared with 34% of patients in the General Medicine Clinic and 59% of patients in the Geriatric Clinic. Correspondingly, weighted adult panel sizes were smaller than unweighted raw panel sizes in Women’s Health Primary Care and Family Medicine Clinics (decreasing in size from 8094 to 6273 and 8079 to 7472, respectively), whereas the weighted panel size was somewhat greater than unweighted at the General Medicine Clinic (9364 unweighted and 10927 weighted) and more than twice as large at the Geriatric Clinic (616 unweighted and 1409 weighted).

**Figure 5:**
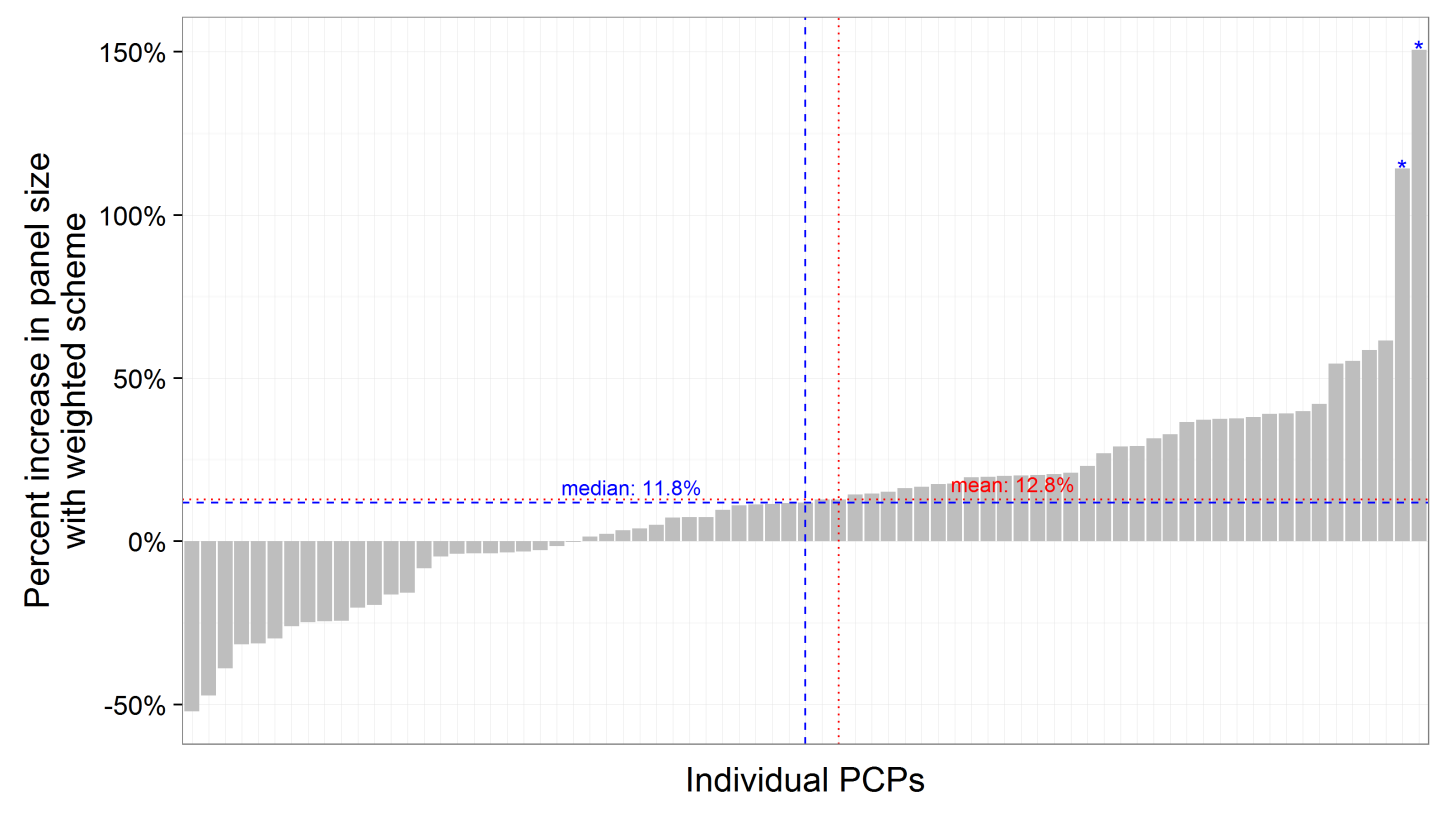
The change of weighted panel size for various primary care providers with more than 150 patients. The panel size increases on average by 12.8%. *These 2 primary care physician (PCPs), who are geriatricians, had a panel size increase of more than 100%.

The relative change in panel size for each individual primary care physician (PCP) is displayed in Figure 5. The relative change in panel size between weighted and unweighted ranged from a relative decrease of 50% to a relative increase of 150%. Two physicians, who care for complex geriatric patients, had weighted panel sizes that were more than double their raw panel sizes. A total of 52% of physicians had a relative change in panel size of 20% or less. Using individual physicians as the unit of analysis, the mean weighted panel size of panel sizes greater than 150 was 12.8% greater than the mean unweighted panel size. The mean change including all panel sizes is 0. Panel sizes that were less than 150 were usually due to physicians working fewer sessions per week (and therefore caring for fewer patients).

**Table 3:** Log-linear model using demographic variables and baseline utilization phenotype to predict subsequent year primary care telephone encounters and office visits among patients in the test set.

Model predictors	Adjusted *R*^2^	AIC^a^
Age-sex^b^	.166	60,780
Payer^c^	.128	61,495
Naïve phenotypes (NP)^d^	.259	57,724
Primary care cluster utilization phenotype (UP)^e^	.330	55,088
Age-sex and payer	.209	59,450
Age-sex, payer, and NP	.343	54,813
Age-sex, payer, and UP	.394	52,769

^a^AIC: Akaike information criterion.

^b^Age-sex bins are categorical variables of the combination of male or female with the following age groups: 18-34, 35-49, 50-64, 65-69, 70-84, and 85-115 years.

^c^Payers are defined as commercial, Medicare or Medicaid, or other.

^d^The naïve phenotype is a categorical variable that is obtained by summing the total number of health care encounters in the baseline year. These values were rank ordered and divided into 7 percentiles.

^e^The utilization phenotype is a categorical variable encoding 1 of the 7 phenotype clusters created by our algorithm.

## Discussion

### Principal Findings

We have described a novel method of using EHR data collected as part of routine care to cluster primary care patients into groups that reflect differences in the primary care work effort required to care for diverse patients. We have demonstrated how this utilization phenotype method can be used to compute weighted panel sizes at the clinic and individual primary care physician (PCP) levels and, by inference, the relative capacity of clinics and PCPs to care for a panel of patients. The utilization phenotype method performed better than other methods in predicting primary care visits in the subsequent year and resulted in weighted panel sizes that differed from unweighted panel sizes at the clinic and individual primary care physician (PCP) levels. The weighting method had face validity when vetted among primary care clinicians caring for patients in the study sample and when comparing results for family medicine, general internal medicine, and geriatrics primary care clinics.

What are the advantages, limitations, and utility of our weighting method? One major strength is that all the data for the algorithm are routinely collected in the EHR. The method takes advantage of the “big data” opportunity afforded by EHRs to use a much richer variety and amount of data to compute weights, compared with traditional methods that primarily rely on a few data elements such as patient demographics and diagnostic codes. All the calculations are transparent (eg, one can inspect the characteristics of patients in each phenotype) and can be rerun easily. The model allows flexibility in assignment of final weights; we assigned weights based on the median number of primary care office visits in a cluster, but the weights could also be determined by consensus or expert opinion or by measurement of median primary care visits for the same clusters in a different health system. Patients are profiled on a single standard, allowing panel sizes to be compared across physicians who care for different populations, such as a geriatrician or a family physician.

We believe that our utilization phenotype approach has conceptual advantages over weighting models that rely on diagnoses coded in EHRs or insurance claims. Our approach does not assume that all patients with a similar diagnosis profile will have similar demands on a health system; instead, a patient’s own activity generates a personalized profile. This allows patients with different disease states, such as interstitial lung disease, obscure gastrointestinal bleeds, or anxiety disorder, to be compared on a single, standardized scale. Reforms in diagnostic coding conventions such as the *International Classification of Diseases, Tenth Revision*, will continue to lack sufficient sensitivity in design and reliability in application to fully capture variation in disease states within diagnostic codes that are meaningful for panel weighting. Moreover, as disease becomes more active or quiescent, the dynamic changes can be reflected in the utilization phenotypes in near real-time. Patients who may have severe diseases but avoid care or use the services of other health care systems are reflected as inactive patients and not weighted highly. If they reengage in care, the new activity will then be reflected in a utilization phenotype. Patients with chronic pain and psychosocial comorbidities often require more frequent touches with the health system than their formal diagnoses would suggest. Rather than inferring primary care work demand from patients’ demographics and diagnoses, our method attempts to more directly estimate work effort. We also captured measures of patient activity that may not be billed, such as secure electronic messaging, medication reconciliation, and care coordination among multiple specialists.

### Limitations

There are several limitations to our algorithm. Our method accepts that observed patterns of service activity reasonably approximate patient demand for primary care work effort. The measure does not distinguish between medically necessary and unnecessary visits, telephone calls, referrals, and other services. A physician who induces inappropriate demand for services would appear in our model to have more complex patients than would a physician who avoids unnecessary services in caring for the same group of patients. However, any system that attempts to measure patient complexity can be gamed, with upcoding diagnostic assessments being a well-known liability for diagnosis-based case mix adjustment methods [12]. To intentionally increase a patient’s complexity by our algorithm, a physician would have to spend more time in care activities, which carries a high opportunity cost. Health systems might consider complementing our panel weighting method with use of other methods to monitor physicians for patterns of wasteful care.

Another important limitation is that because the panel weighting is normalized within our system to make the total weighted patient count equal to the unweighted count, the method cannot be easily used to compare the relative primary care work demand of primary care patients in our system with that of patients in another system. If additional systems using the same EHR vendor begin to use this model and are willing to collaborate on the final weighting steps, cross-system comparisons may be possible. Our model also does not answer the question of what the “right” weighted panel size should be for a given health system. A final limitation is that our method does not as yet include children. We are developing a similar algorithm to apply to this population.

### Conclusions

Our panel weighting model may be useful when implementing a variety of health system policies related to primary care empanelment. One fundamental element of empanelment is matching capacity with demand, which requires determining whether a primary care clinic or physician is “underempaneled” relative to a benchmark goal and therefore should accept new patients. It is difficult for an organization to achieve primary care physician (PCP) buy-in for regulation of panel size without a credible method of patient weighting to address physician concerns that raw counts do not accurately reflect panel variation. Weighted panel measurement may also assist health systems in prioritizing support staff to clinics and physicians with the highest work demand.

In summary, we have reported a novel clustering approach for primary care patients using routinely collected EHR data that can be used to create weighted panel sizes and dynamically load-balance PCPs and clinics. Our use of physician review of the clusters and predictive modeling suggests the algorithm identifies clinically meaningful phenotypes that are correlated with future primary care utilization. As health delivery and payment models shift to emphasize a population health orientation, weighting of primary care panels will assume greater importance for aligning primary care capacity and resources with variation in primary care work effort needed to care for different types of patients. Our weighting method attempts to capture this variation across patients in primary care work demand and may be implemented into population health analytic processes in a manner that allows near real-time calculation of weights in response to dynamic changes in patients’ clinical activity.

## Multimedia Appendix 1

The absolute and change of within-group sum of squares with each additional cluster. The change in within-group sum of squares starts to level off at 4 groups.

## Multimedia Appendix 2

Patient Characteristics of Each Utilization Phenotype in the Training Set (n=24,324).

## Multimedia Appendix 3

Patient Characteristics of each Primary Care Work Cluster in the Training Set (n=24,324).

## Multimedia Appendix 4

Log-Linear model of primary care office visits (without telephone visits) based on demographic variables and baseline utilization phenotype.

## Multimedia Appendix 5

The unweighted and weighted patient counts across the primary care work clusters.

## Abbreviations

AIC: Akaike information criterion
EHR: electronic health record
PCP: primary care physician
UCSF Health: University of California, San Francisco health system
