# Supplementary figures and images for: Weighting Primary Care Patient Panel Size: A Novel Electronic Health Record-Derived Measure Using Machine Learning
Source: JMIR Med Inform. 2016 Oct 14;4(4):e29. doi: 10.2196/medinform.6530 (PMC5086026; doi:10.2196/medinform.6530)

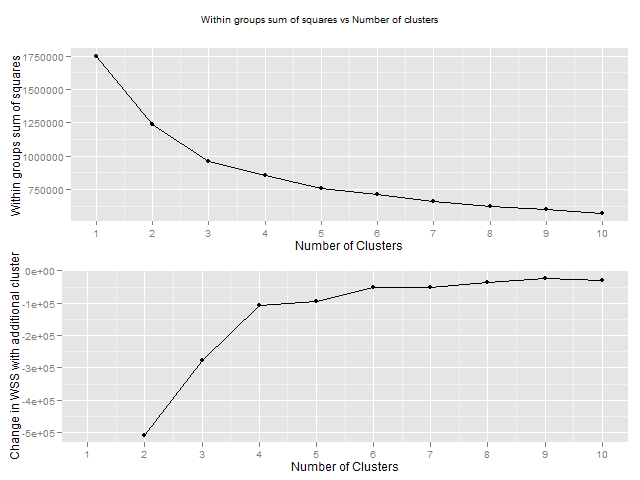

Supplement: Supplementary file 1 [file medinform_v4i4e29_app1.png]

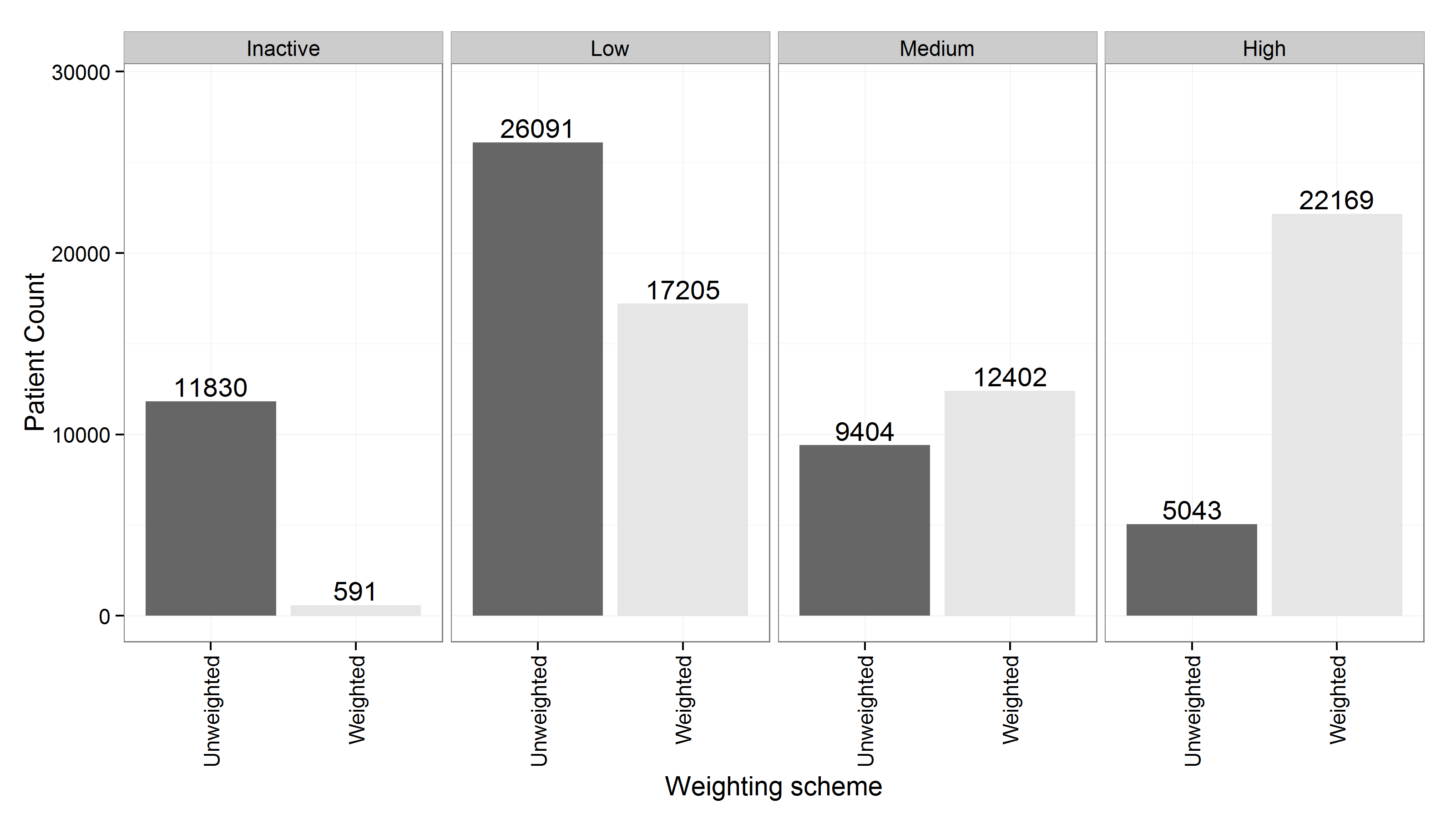

Supplement: Supplementary file 5 [file medinform_v4i4e29_app5.png]
