# Supplementary material for: Weighting Primary Care Patient Panel Size: A Novel Electronic Health Record-Derived Measure Using Machine Learning
Source: JMIR Med Inform. 2016 Oct 14;4(4):e29. doi: 10.2196/medinform.6530 (PMC5086026; doi:10.2196/medinform.6530)
Supplement: Supplementary file 2 [file medinform_v4i4e29_app2.pdf]

Multimedia Appendix 1. Patient characteristics of each utilization phenotype in the training set (n=24,324).

| Characteristics | Utilization phenotype | | | | | | | | |
| --- | --- | --- | --- | --- | --- | --- | --- | --- | --- |
| | Inactive | A | B | C | D | E | F | G | Total sample |
| Size of group | 3986 | 5343 | 6991 | 3000 | 2452 | 2082 | 430 | 40 | 24,324 |
| Age, years, mean (SD) | 41.9 (17.3) | 47.7 (14.7) | 53.7 (16.8) | 56.6 (16.4) | 59.9 (17.3) | 65.1 (16.8) | 67.4 (16.3) | 60.5 (14.4) | 52.7 (17.9) |
| Male, n (%) | 1551 (38.9) | 2057 (38.5) | 2678 (38.3) | 1083 (36.1) | 922 (37.6) | 716 (34.4) | 158 (36.7) | 8 (20) | 9170 (37.7) |
| White, n (%) | 1814 (45.5) | 2875 (53.8) | 3293 (47.1) | 1635 (54.5) | 1324 (54.0) | 799 (38.4) | 191 (44.4) | 14 (35) | 11,943 (49.1) |
| Asian, n (%) | 694 (17.4) | 1095 (20.5) | 1734 (24.8) | 675 (22.5) | 596 (24.3) | 525 (25.2) | 76 (18) | 4 (10) | 5400 (22.2) |
| Black, n (%) | 379 (9.5) | 289 (5.4) | 587 (8.4) | 222 (7.4) | 184 (7.5) | 385 (18.5) | 102 (23.7) | 17 (43) | 2165 (8.9) |
| Commercial, n (%) | 2738 (68.7) | 4266 (79.9) | 4348 (62.2) | 1731 (57.7) | 1113 (45.4) | 431 (20.7) | 26 (6) | 3 (8) | 14,665 (60.3) |
| Medicare or Medicaid, n (%) | 1068 (26.8) | 992 (18.6) | 2545 (36.4) | 1245 (41.5) | 1324 (54.0) | 1628 (78.2) | 402 (93.5) | 37 (93) | 9219 (38.0) |
| Other payer, n (%) | 180 (4.5) | 85 (2) | 98 (1) | 24 (1) | 15 (1) | 23 (1) | 2 (1) | N/A[a] | 440 (1.8) |
| Active medications at PCP visit, mean (SD) | 0 (0) | 2.3 (2.9) | 5 (3.6) | 5.5 (4.3) | 8.1 (6) | 11 (5) | 15.7 (6.1) | 16.2 (9.3) | 4.7 (5.2) |
| Primary care visits, mean (SD) | 0 (0) | 0.7 (0.5) | 2.6 (1.3) | 2.1 (1.4) | 2.9 (2.3) | 7 (2.8) | 11.5 (4.5) | 33.2 (10) | 2.3 (3) |
| Weighted primary care visits, mean (SD) | 0 (0) | 0.7 (0.6) | 3.2 (1.8) | 2.8 (1.9) | 4.3 (3.7) | 10.7 (4.4) | 19.1 (7.8) | 53.1 (15.6) | 3.2 (4.7) |
| No-show visits, mean (SD) | 0.1 (0.4) | 0.2 (0.7) | 0.5 (1) | 0.6 (1.2) | 1.4 (2.1) | 1.8 (2.4) | 4.3 (4.9) | 6.2 (5.3) | 0.7 (1.6) |
| Urgent care visits, mean (SD) | 0 (0) | 0.1 (0.5) | 0.2 (0.5) | 0.2 (0.6) | 0.2 (0.6) | 0.2 (0.7) | 0.5 (1.2) | 1.2 (2.4) | 0.1 (0.5) |

| Characteristics | Utilization phenotype | | | | | | | | |
|---|---|---|---|---|---|---|---|---|---|
| | Inactive | A | B | C | D | E | F | G | Total sample |
| Telephone encounters, mean (SD) | 0 (0) | 0.4 (0.7) | 1.7 (1.8) | 1.4 (1.6) | 2.3 (2.5) | 5.9 (3.8) | 19.4 (10.3) | 18.5 (22.5) | 1.9 (3.8) |
| Emergency department visits, mean (SD) | 0 (0) | 0 (0) | 0.2 (0.5) | 0.2 (0.5) | 0.3 (0.7) | 0.5 (1) | 1.6 (2.9) | 1.8 (2.1) | 0.2 (0.7) |
| Emergent hospitalizations, mean (SD) | 0 (0) | 0 (0) | 0 (0.2) | 0 (0.3) | 0.1 (0.5) | 0.2 (0.5) | 0.9 (1.7) | 0.9 (1.5) | 0.1 (0.4) |
| Elective hospitalizations, mean (SD) | 0 (0) | 0 (0) | 0 (0) | 0 (0.2) | 0.1 (0.3) | 0 (0.2) | 0.1 (0.4) | 0 (0.2) | 0 (0.1) |
| Specialist visits (capped), mean (SD) | 0 (0) | 1 (1.2) | 1 (1) | 5.5 (1.4) | 14 (5.3) | 4.4 (3.3) | 11.5 (8.2) | 7.6 (9.2) | 3.2 (4.9) |
| Infusion visits, mean (SD) | 0 (0) | 0 (0.5) | 0 (0.4) | 0.1 (1) | 0.7 (4.1) | 0.1 (2.2) | 0.1 (1.1) | 0 (0.2) | 0.1 (1.5) |
| Transfusion visits, mean (SD) | 0 (0) | 0 (0.8) | 0 (0.2) | 0.1 (0.8) | 0.4 (2.6) | 0 (0.6) | 0.5 (3.3) | 0.2 (1.3) | 0.1 (1.1) |
| Radiology or procedure visits, mean (SD) | 0 (0) | 0.4 (0.8) | 0.6 (1) | 1.2 (1.4) | 2.2 (2.6) | 1.5 (1.7) | 2.7 (3) | 2.5 (2.9) | 0.8 (1.5) |
| Secure electronic messages to patient, mean (SD) | 0 (0) | 0.7 (1.4) | 2.3 (4.3) | 4 (6.4) | 6.8 (11.1) | 3.4 (7.8) | 5.6 (14.6) | 5 (14.5) | 2.4 (6.1) |
| Secure electronic messages from patient, mean (SD) | 0 (0) | 0.9 (1.8) | 2.8 (5.3) | 5 (8.2) | 8.9 (15) | 4.4 (10.4) | 8.1 (22.3) | 8.6 (25.5) | 3.1 (8.1) |

[a]N/A: not applicable.
