# Supplementary material for: Weighting Primary Care Patient Panel Size: A Novel Electronic Health Record-Derived Measure Using Machine Learning
Source: JMIR Med Inform. 2016 Oct 14;4(4):e29. doi: 10.2196/medinform.6530 (PMC5086026; doi:10.2196/medinform.6530)
Supplement: Supplementary file 3 [file medinform_v4i4e29_app3.pdf]

Multimedia Appendix 2: Patient Characteristics of each Primary Care Work Cluster in the Training Set (n=24,324)

	Primary Care Work Cluster				
	Inactive	Low	Medium	High	Total sample
Size of group	3986	12003	5534	2801	24,324
Age, mean (SD)	41.9 (17.3)	51 (16.2)	57.8 (16.9)	65 (16.6)	52.7 (17.9)
Male, n (%)	1551 (38.9)	4621 (38.5)	2025 (36.6)	980 (35)	(37.7)
White, n (%)	1814 (45.5)	5941 (49.5)	2999 (54.2)	1196 (42.7)	(49.1)
Asian, n (%)	694 (17.4)	2797 (23.3)	1284 (23.2)	633 (22.6)	(22.2)
Black, n (%)	379 (9.5)	864 (7.2)	404 (7.3)	513 (18.3)	(8.9)
Commercial, n (%)	2738 (68.7)	8390 (69.9)	2944 (53.2)	585 (20.9)	14,665 (60.3)
Medicare or Medicaid, n (%)	1068 (26.8)	3433 (28.6)	2546 (46.0)	2191 (78.2)	9219 (38.0)
Other, n (%)	180 (4.5)	180 (1.5)	44 (1)	25 (1)	440 (1.8)
Active medications at PCP visit, mean (SD)	0 (0)	3.8 (3.6)	6.5 (5.2)	11.7 (5.7)	4.7 (5.2)
Primary care visits, mean (SD)	0 (0)	1.8 (1.4)	2.4 (1.8)	7.7 (5)	2.3 (3)
Weighted primary care visits, mean (SD)	0 (0)	2.1 (1.8)	3.4 (2.9)	12.1 (8.1)	3.2 (4.7)
No-show visits, mean (SD)	0.1 (0.4)	0.4 (0.9)	0.9 (1.7)	2.2 (3.1)	0.7 (1.6)
Urgent care visits, mean (SD)	0 (0)	0.1 (0.5)	0.2 (0.6)	0.3 (0.9)	0.1 (0.5)
Telephone encounters, mean (SD)	0 (0)	1.1 (1.5)	1.8 (2.1)	7.8 (7.9)	1.9 (3.8)

	Primary Care Work Cluster				
	Inactive	Low	Medium	High	Total sample
Emergency department visits, mean (SD)	0 (0)	0.1 (0.4)	0.2 (0.6)	0.6 (1.5)	0.2 (0.7)
Emergent hospitalizations, mean (SD)	0 (0)	0 (0.1)	0.1 (0.4)	0.3 (0.9)	0.1 (0.4)
Routine hospitalizations, mean (SD)	0 (0)	0 (0)	0 (0.2)	0.1 (0.2)	0 (0.1)
Specialist visits (capped), mean (SD)	0 (0)	1 (1.1)	8.7 (5.6)	6.4 (6)	3.2 (4.9)
Infusion visits, mean (SD)	0 (0)	0 (0.5)	0.3 (2.6)	0.2 (2.6)	0.1 (1.5)
Transfusion visits, mean (SD)	0 (0)	0 (0.5)	0.2 (1.7)	0.2 (1.6)	0.1 (1.1)
Radiology or procedure visits, mean (SD)	0 (0)	0.5 (0.9)	1.5 (1.9)	1.9 (2.3)	0.8 (1.5)
Secure electronic messages to patient, mean (SD)	0 (0)	1.2 (2.3)	4.5 (6.1)	6.6 (13.5)	2.4 (6.1)
Secure electronic messages from patient, mean (SD)	0 (0)	1.5 (2.7)	5.8 (7.7)	8.9 (18.8)	3.1 (8.1)
