# Supplementary material for: Weighting Primary Care Patient Panel Size: A Novel Electronic Health Record-Derived Measure Using Machine Learning
Source: JMIR Med Inform. 2016 Oct 14;4(4):e29. doi: 10.2196/medinform.6530 (PMC5086026; doi:10.2196/medinform.6530)
Supplement: Supplementary file 4 [file medinform_v4i4e29_app4.pdf]

Multimedia Appendix 4: Log-Linear model of primary care office visits (without telephone visits) based on demographic variables and baseline utilization phenotype

Model predictors	Adjusted R^2	AIC[a]
Age-sex[b]	.135	49,638
Payer[c]	.112	49,989
Naïve phenotypes (NP)[d]	.241	46,261
Utilization phenotype (UP)[e]	.296	44,344
Age-sex and payer	.173	48,505
Age-sex, payer, and NP	.306	44,147
Age-sex, payer, and UP	.344	42,695

[a]Akaike Information Criterion
[b]Age-sex bins are categorical variables of the combination of male or female with the following age groups: 18-34, 35-49, 50-64, 65-69, 70-84,85-115.
[c]Payer were defined as commercial, Medicare/Medical, or Other
[d]The naïve phenotype is a categorical variable that was obtained by summing to the total number of healthcare encounters in the baseline year. These values were rank ordered and divided into seven percentiles
[e]The utilization phenotype is a categorical variable encoding one of the seven phenotypes clusters created by our algorithm.
